# Replication of a distinct psoriatic arthritis risk variant at the *IL23R* locus

**DOI:** 10.1136/annrheumdis-2016-209290

**Published:** 2016-03-25

**Authors:** Ashley Budu-Aggrey, John Bowes, Sabine Loehr, Steffen Uebe, Maria I Zervou, Philip Helliwell, Anthony W Ryan, David Kane, Eleanor Korendowych, Emiliano Giardina, Jonathan Packham, Ross McManus, Oliver FitzGerald, Neil McHugh, Frank Behrens, Harald Burkhardt, Ulrike Huffmeier, Pauline Ho, Javier Martin, Santos Castañeda, George Goulielmos, Andre Reis, Anne Barton

**Affiliations:** 1 Arthritis Research UK Centre for Genetics and Genomics, Centre for Musculoskeletal Research, The University of Manchester, UK; 2 NIHR Manchester Musculoskeletal Biomedical Research Unit, Central Manchester Foundation Trust and University of Manchester, Manchester Academy of Health Sciences, Manchester, UK; 3 Institute of Human Genetics, Friedrich-Alexander-Universität Erlangen-Nürnberg, Erlangen, Germany; 4 Laboratory of Molecular Medicine and Human Genetics, Department of Internal Medicine, University of Crete, Heraklion, Greece; 5 NIHR-Leeds Musculoskeletal Biomedical Research Unit Leeds Institute of Rheumatic and Musculoskeletal Medicine, University of Leeds, Leeds, UK; 6 Department of Clinical Medicine, Institute of Molecular Medicine, Trinity College Dublin, Dublin, Ireland; 7 Department of Rheumatology, Adelaide and Meath Hospital and Trinity College Dublin, Ireland; 8 Royal National Hospital for Rheumatic Diseases and Department Pharmacy and Pharmacology, University of Bath, Bath, UK; 9 Department of Biomedicine and Prevention, University of Rome ‘Tor Vergata’ and Laboratory of Molecular Genetics UILDM, Fondazione Santa Lucia IRCCS, Rome, Italy; 10 Rheumatology Department, Haywood Hospital, Health Services Research Unit, Institute of Science and Technology in Medicine, Keele University, Stoke on Trent, UK; 11 Department of Rheumatology, St. Vincent's University Hospital, UCD School of Medicine and Medical Sciences and Conway Institute of Biomolecular and Biomedical Research, University College Dublin, Dublin, Ireland; 12 Division of Rheumatology and Fraunhofer IME-Project-Group Translational Medicine and Pharmacology, Goethe University, Frankfurt, Germany; 13 The Kellgren Centre for Rheumatology, Central Manchester Foundation Trust, NIHR Manchester Biomedical Research Centre, Manchester, UK; 14 CSIC, Instituto de Parasitologia y Biomedicina Lopez-Neyra, Granada, Spain; 15 Department of Rheumatology, Hospital La Princesa, IIS-IPrincesa, Madrid, Spain

**Keywords:** Gene Polymorphism, Psoriatic Arthritis, Arthritis

Psoriatic arthritis (PsA) is a chronic inflammatory arthritis associated with the presence of psoriasis. Although the majority of PsA genetic risk loci identified also confer risk for psoriasis, the difference in heritability between the two diseases suggests that there remain uncovered risk loci that are associated with PsA but not psoriasis (herein called PsA-specific loci).^1^ Here we present an independent replication of a PsA-specific association to rs12044149 at the well-established psoriasis risk locus, *IL23R*, and confirm its independence of the previously reported psoriasis single-nucleotide polymorphism (SNP); rs9988642.^2^ ^3^

Following quality control, genotype data were available for Spanish, Cretan, German and UK PsA cases (914) and controls (6945), independent of those tested previously^2^ for rs12044149 and rs9988642. All PsA case subjects were recruited from rheumatology clinics and were diagnosed by a rheumatologist. Genotype data were generated using the Life Technologies TaqMan chemistry on the QuantStudio platform. Data were also available for samples that had been previously genotyped. Case–control association testing was performed separately for each dataset using logistic regression in PLINK. This was followed by a meta-analysis of the summary statistics, using an inverse-variance fixed-effects model (table 1). The association of rs12044149 with PsA was replicated (p=4.03×10^−6^) and remained significant after including rs9988642 as a covariate (p_cond_=4.86×10^−6^). Meta-analysis of this data with that of our previous Immunochip study reached genome-wide significance (p_meta_=4.76×10^−20^) in 2876 cases and 15 868 controls (table 1). For the previously reported psoriasis variant, rs9988642,^3^ only modest association was found with PsA in the replication meta-analysis (p=0.04), and did not reach genome-wide significance upon meta-analysis of the combined PsA dataset (p=4.61×10^−4^) (table 1).

**Table 1:** Summary statistics for Immunochip, replication and meta-analysis of PsA and psoriasis variants at *IL23R*

		Replication meta-analysis				Immunochip		Meta-analysis (All)			
SNP	Minor/major allele	p Value	OR	I^2^	Q	p Value	OR	p Value	OR	I^2^	Q
rs12044149	T/G	4.03E-06	1.33	10.15	0.34	2.25E-15	1.36	4.76E-20	1.35	0	0.49
rs9988642	C/T	0.04	0.78	34.51	0.21	4.51E-03	0.81	4.61E-04	0.80	13.76	0.33

I^2^, heterogeneity index for ORs; PsA, psoriatic arthritis; Q, Cochrane's Q statistic for heterogeneity of ORs; SNP, single-nucleotide polymorphism.

We investigated differences between PsA and psoriasis by combining our data with a subset of the psoriasis WTCCC2 study,^4^ excluding patients with known PsA (1784 psoriasis cases, 5175 controls). Here, rs9988642 was significantly associated with psoriasis (p=1.0×10^−7^) and remained so after conditioning for rs12044149 (p_cond_=1.63×10^−5^). The effect estimates of rs12044149 for PsA and psoriasis were significantly different (p=2.0×10^−3^), when tested using multinomial logistic regression in Stata. Direct comparison of the PsA and psoriasis genotypes revealed a significant association with an increased risk of PsA with rs12044149 (p=4.52×10^−4^, OR=1.3). While we cannot exclude the possibility of undiagnosed PsA cases in the psoriasis group, their inclusion would have biased the results to the null hypothesis of no difference in the association statistics between PsA and psoriasis. These results support previously reported evidence that the association signals for the *IL23R* variants rs12044149 and rs9988642 are independent of each other and that the association to rs12044149 is specific to PsA.^2^ ^5^

**Figure 1:**
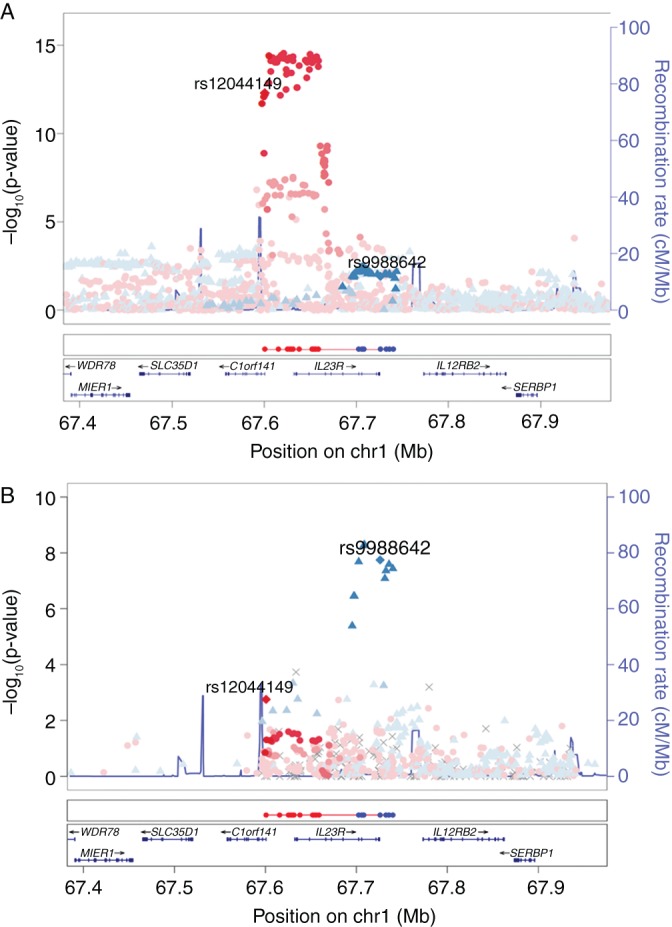
(A) Association of *IL23R* variants in psoriatic arthritis (PsA) Immunochip study (1962 cases, 8923 controls). (B) Association of *IL23R* variants in psoriasis Immunochip study (2997 cases, 9183 controls). Credible single-nucleotide polymorphism sets are depicted below each plot for the rs12044149 (red) and rs9988642 (blue) association signals.

A Bayesian refinement method was applied to define credible SNP sets based on each effect, as described previously.^2^ These were localised to regulatory features using data from the Roadmap Epigenomics Project^6^ and the online genetic and epigenetic finemapping data portal^7^ (figure 1). Credible SNPs (n=13) for the PsA-specific associated SNP mapped to promoter and enhancer regions within memory CD8^+^ T cells, which we have previously reported to be critical for PsA.^2^ By contrast, credible SNPs based on the psoriasis association (n=7) did not overlap with regulatory elements and one, rs11209026, was found to cause a missense mutation, resulting in an Arg381Gln substitution.^8^ This could suggest the involvement of different functional mechanisms for the PsA and psoriasis associations.

In conclusion, we have replicated a PsA-specific association at the *IL23R* locus within an independent population, and confirm that the association with rs12044149 is distinct from the psoriasis *IL23R* variant, rs9988642. Currently, five robust PsA-specific loci have been identified, including HLA-B,^2^ chromosome 5q31,^2^ *PTPN22*^9^ and *TNFAIP3.*^5^ Such associations can provide potential clinical benefits in disease-risk estimation and linking genetics with therapeutic targets.
